# Effect of Residual Stress and Microstructure on the Fatigue Crack Growth Behavior of Aluminum Friction Stir Welded Joints

**DOI:** 10.3390/ma17020385

**Published:** 2024-01-12

**Authors:** Yanning Guo, Peiyao Li

**Affiliations:** 1 School of Civil Engineering, Xi’an University of Architecture and Technology, Xi’an 710055, China; 2 School of Aeronautics, Northwestern Polytechnical University, Xi’an 710072, China

**Keywords:** aluminum alloys, friction stir welding, fatigue crack growth, residual stress, microstructure

## Abstract

Friction stir welding (FSW) has been adopted in the aerospace industry for fabricating structural alloys due to the low melting point and high thermal conductivity of aviation aluminum alloys. However, welding residual stresses can lead to secondary deformation in friction stir welded (FSWed) structures. Additionally, microstructural characteristics impact the crack growth rates and directions in these structures. Therefore, it is necessary to investigate the effects of residual stress and microstructure on the fatigue responses of FSWed joints. In this paper, we studied the fatigue crack growth behavior of two homogeneous and dissimilar FSWed joints with varying welding parameters, namely 2024-T3 and 7075-T6. The residual stresses were measured with the X-ray diffraction method. The dislocations and precipitates in different zones of the FSWed joints were analyzed via transmission electron microscopy (TEM). The results demonstrated that the residual stress significantly affected the fatigue crack growth rate and direction; the tensile residual stress promoted fatigue crack growth and offset the decrease in the fatigue crack growth rate that occurred due to grain refinement. The results of the microstructural analysis indicated that dislocation density and sliding resistance increased with the decrease in rotational speed and led to a decreased rate of fatigue crack propagation.

## 1. Introduction

AA 2xxx and AA 7xxx aluminum alloys are widely used in aircraft structures due to their low density, high strength, and high stiffness [1]. Specifically, the 2024-T3 alloy is primarily employed in the fuselage for the rivets, skin, wings, skeleton, etc. The 7075-T6 alloy serves as the essential material in wing spars, baffle frames, and wing panels. However, traditional welding methods cause several defects in the 2024-T3 and 7075-T6 alloys [2]. On the other hand, friction stir welding (FSW) stands out as a typical solid-state joining technology, which is capable of fabricating high-quality welded joints with minimal welding deformation [3,4]. Therefore, FSW is being increasingly adopted in industries such as aerospace, shipping, railway, electronics, and others [5,6].

During the welding process, changes in local temperature and deformation induce plastic deformation, which occurs in the welded zone (WZ) and thereby leads to the formation of residual stress, which is a self-balancing system that is not dependent on external loads [3,7]. The residual stress and the external loads can result in secondary deformations, as well as the redistribution of residual stress, which significantly influences the integrity and safety of welded structures [8,9,10]. Previous studies have shown that the maximum residual stress in the longitudinal direction (LD) is generated in the heat-affected zone (HAZ), while the minimum compressive residual stress originates on the advancing side just beyond the WZ [11,12]. Furthermore, another study indicated that in the 2024/7075 dissimilar welded joints, the longitudinal residual stress on the 7075 side was higher than that on the 2024 side [13]. This is related to the position within the welded joint, as the heat input on the advancing side is relatively higher than that on the retreating side. Additionally, compared to the 2024 alloy, 7075 aluminum exhibits higher mechanical properties, including yield strength and hardness.

Due to the strong local thermal coupling effect in the FSW process, the microstructure of the welded joint becomes diversified and non-uniform [14,15]. The WZ, which is composed of fine equiaxed grains experiences the highest thermal cycle and the most intense plastic deformation during the welding process, causing dynamic recrystallization in this region [16]. In the thermo-mechanically affected zone (TMAZ), plastic deformation induces the formation of several dislocations within the crystal structure. Additionally, the increase in temperature leads to dynamic recovery, thereby resulting in the formation of a large number of sub-grains [17]. A previous study demonstrated that the HAZ exclusively underwent the thermal cycle that results in a partial coarsening of the grains, which typically leads to a morphology that closely resembles that of the base metal [18].

Previous studies have also demonstrated that the fatigue crack growth rate in FSWed joints is influenced by the interaction between residual stress distribution and the microstructure [19,20]. The high level of compressive residual stress near the welded zone can induce crack closure effects, while tensile stress increases the effective stress intensity factor range [21,22]. Fratini et al. [23,24] noted that stress relief did not alter the hardness and microstructural properties of FSWed structures, but the closure phenomena resulting from residual stress did affect the growth rates. Ilman et al. [25] stated that the effect of the microstructure on the fatigue crack growth rate was not significant when compared to residual stress. However, in the study by Anderson-Wedge et al. [26], the changes in crack growth were attributed to the depletion of strengthening precipitates in thermally affected zones rather than to the residual stresses.

Considering the varying conclusions from previous studies, this paper aims to investigate the coupling effect between residual stress and microstructure on the fatigue crack growth behavior of 2024 and 7075 aluminum alloys. The residual stresses were measured with the X-ray diffraction method, and the microstructure was observed with TEM. The finite element model was built using ANSYS and FRANC3D to calculate the fatigue crack growth rate, and the results were compared with those of the subsequent experiments.

## 2. Experimental Section

Rectangular plates of 2024-T3 and 7075-T6 alloys that were 87.5 mm × 400 mm × 8 mm in size were joined using the FSW-RL31-010 machine (Beijing FSW Technology Co., Ltd., Beijing, China) at Northwestern Polytechnical University in Xi’an, China. The shoulder diameter was 20 mm, and the probe diameters were 5 mm and 7.5 mm. Table 1 displays the chemical compositions of the 2024-T3 and 7075-T6 alloys, which were produced by Shanghai Miandi Metal Group Co., Ltd. (Shanghai, China) The welding parameters are shown in Table 2.

## 3. Residual Stress Measurement

**Figure 1:**
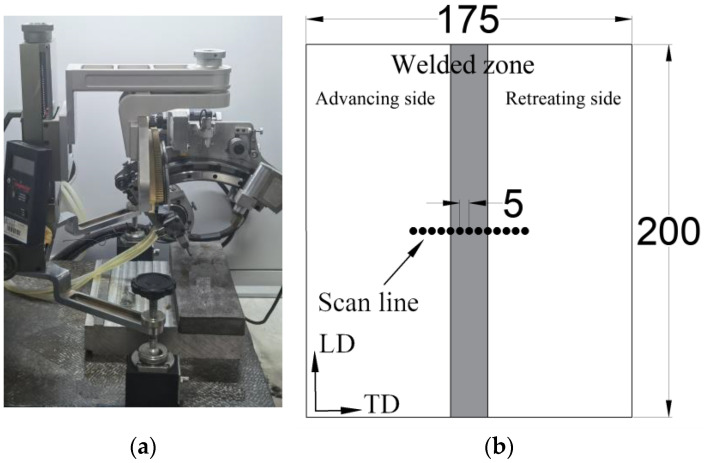
Residual stress measurement: (**a**) X-ray diffraction method; (**b**) scan line (mm).

The residual stress distribution of the 2024 specimen (400 rpm-150 mm/min) was analyzed using the X-350A X-ray diffraction setup at Xi’an Jiaotong University in Xi’an, China (Figure 1a). The stress distributions in both the longitudinal direction (LD) and the transverse direction (TD) were measured separately. The scan line is illustrated in Figure 1b.

**Figure 2:**
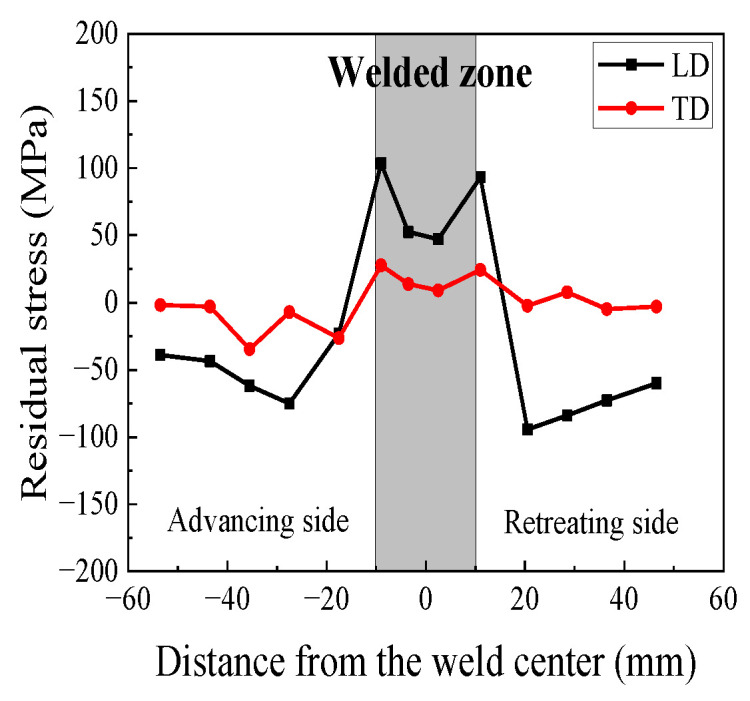
Results of residual stress measurement.

The residual stress profile of the 2024 FSWed specimen (400 rpm-150 mm/min) is shown in Figure 2. The LD stress profile exhibits a “double peak” near the welded zone, with a peak stress of 103.2 MPa on the advancing side and a maximum stress of 93.5 MPa on the retreating side. This difference arises due to the comparatively higher heat input on the advancing side. In the TD, the residual stress values are relatively uniform, with a peak stress of 27.65 MPa on the advancing side.

## 4. Fatigue Crack Growth Experiment

**Figure 3:**
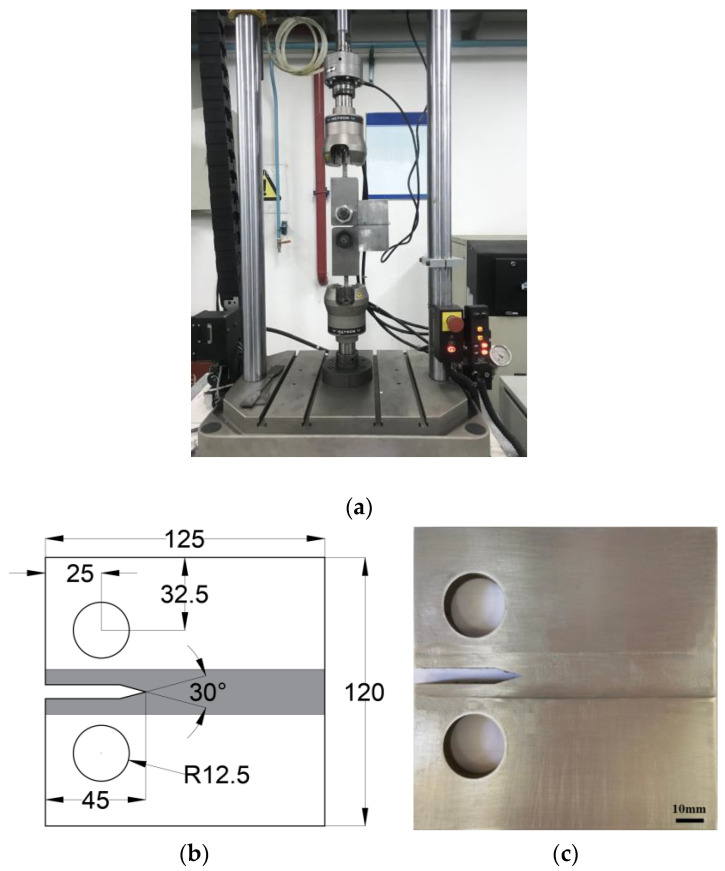
(**a**) Fatigue crack growth experiment; (**b**) size of the C(T) specimens (mm); (**c**) C(T) specimen.

The fatigue crack growth experiments in this study were conducted using an INSTRON 810 testing machine at Northwestern Polytechnical University, Xi’an, China, with a maximum loading force of 26 kN (Figure 3a). This setup ensured symmetrical load distribution with a load error of less than ±1% and minimal variation in the indicating value. The testing machine was equipped with an accurate counting device to meet the testing requirements. The compact tension (C(T)) specimens were machined according to the ASTM-647 standard [27]; the size of the specimen is shown in Figure 3b. It can be seen that the cracks in the C(T) specimens were parallel to the welded joints. To facilitate fixture fixation and crack growth observation, the surfaces of the specimen were polished before the experiment (Figure 3c). Fatigue tests were performed at R = 0.1 for all the C(T) specimens, with a fatigue load frequency of 20 Hz. Load ranges of 6.21 kN for the 2024 and 7075 homogeneous specimens, 8.04 kN for the 2024/7075 dissimilar specimens, and 3.26 kN for the base metal were applied.

**Figure 4:**
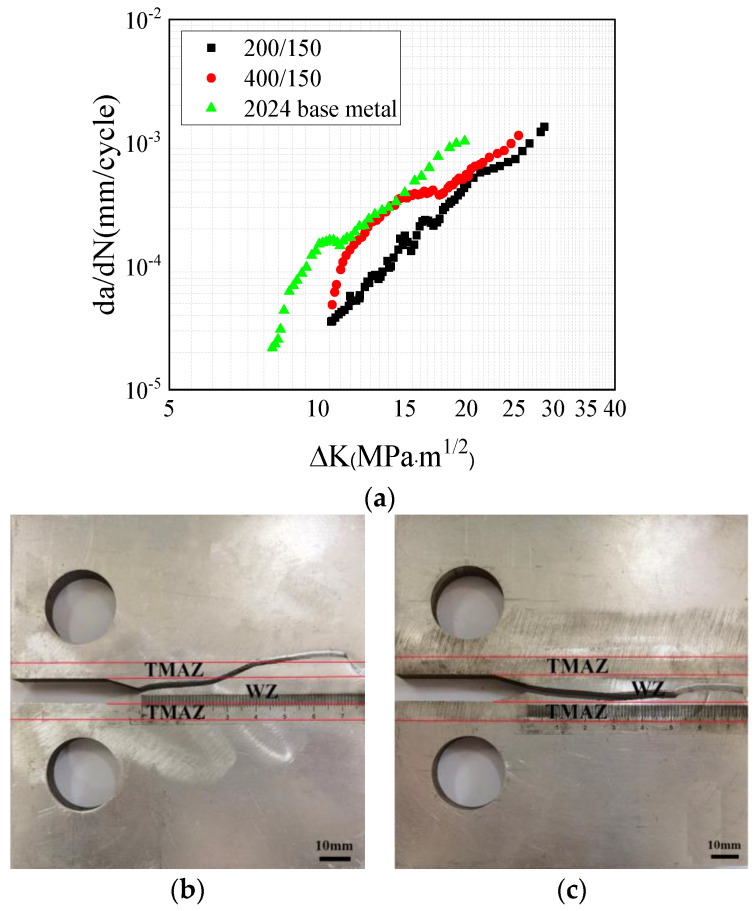
Fatigue crack growth results of the 2024 specimen: (**a**) crack growth rates; (**b**) crack growth path at 200 rpm; (**c**) crack growth path at 400 rpm.

The results of the fatigue crack growth rate experiment of the 2024 homogeneous FSWed specimens with different rotational speeds are presented in Figure 4. It can be seen that the crack growth rate of the 2024 base metal is higher than that of the welded specimen. Under the same welding speed, the fatigue crack growth rate of the 400 rpm specimen is higher than that the of 200 rpm specimen. As seen in Figure 4b, the crack path of 200 rpm was deflected towards the loading hole. With further crack propagation, the crack crossed through the WZ into the thermo-mechanically affected zone (TMAZ). Figure 4c shows that the crack growth path of the 400 rpm specimen remained within the WZ. According to previous studies on the dynamic tensile properties of the WZ, the increase in rational speed reduces the size of the particles in the WZ and makes them more uniform [28]. This enhances the mechanical properties of the WZ and reduces the fatigue crack growth rate in this region. Therefore, it can be concluded that tensile longitudinal residual stress promotes the crack growth and that it offsets the decrease in fatigue crack growth rate by grain refinement.

**Figure 5:**
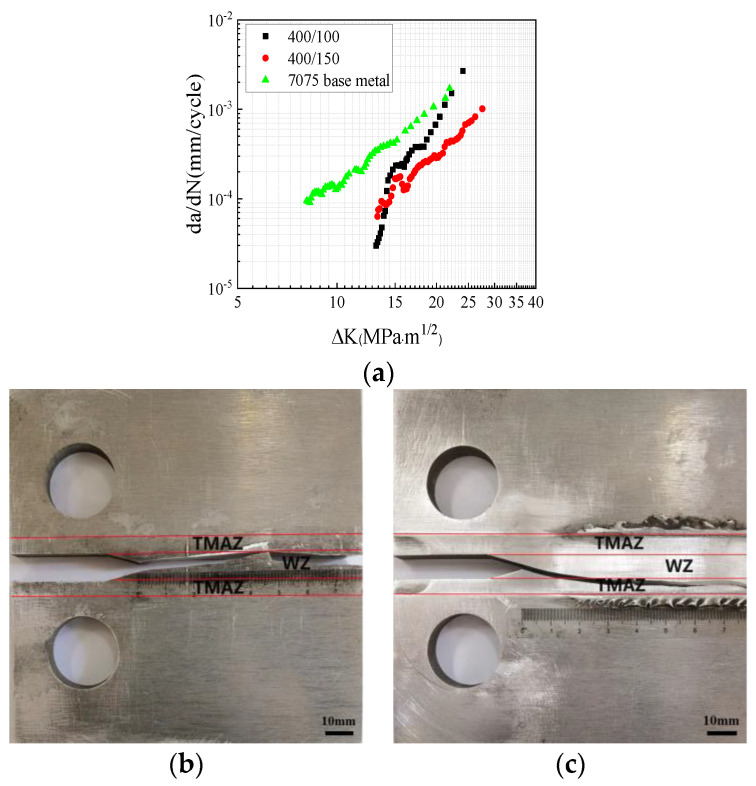
Fatigue crack growth results of the 7075 specimen: (**a**) crack growth rates; (**b**) crack growth path at 100 mm/min; (**c**) crack growth path at 150 mm/min.

Figure 5a displays the fatigue crack growth results of the 7075 homogeneous FSWed specimens with different welding speeds. It is evident that welding speed exerts a significant influence on the crack growth rate. At the initial stage, the crack growth rate of the 7075 base metal was much higher than that of the FSWed specimen. Notably, the fatigue crack growth rate of the specimen with a welding speed of 100 mm/min was higher than that of the specimen with a speed of 150 mm/min. In a previous study, it was demonstrated that the mechanical properties of the 100 mm/min and 150 mm/min specimens were similar [29]; however, the longitudinal residual stress of the 100 mm/min specimen was higher than that of the 150 mm/min specimen. Consequently, the reduction in longitudinal residual stress contributed to the lower fatigue crack growth rate.

**Figure 6:**
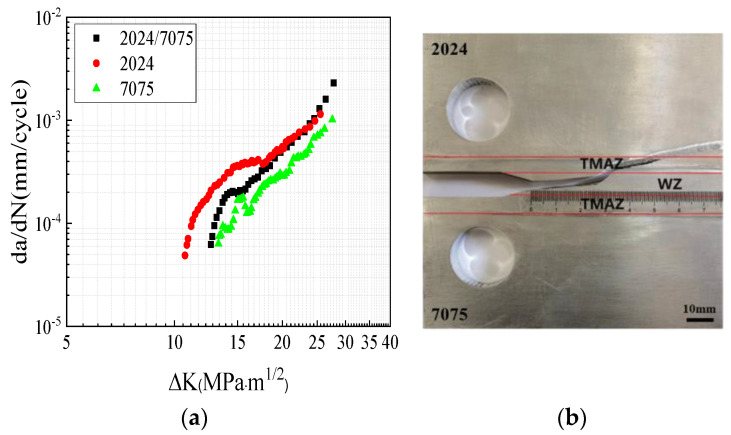
Fatigue crack growth results of the 2024/7075 dissimilar specimen: (**a**) crack growth rates; (**b**) crack growth path.

Figure 6a shows the fatigue crack growth rate curve of the 2024/7075 dissimilar FSWed specimen with a welding speed of 150 mm/min and rational speed of 400 rpm. It was compared with the 2024 and 7075 homogeneous FSWed specimens under the same welding parameters. The results indicated that, during the initial stage of crack growth, the fatigue crack growth rate of the 2024/7075 dissimilar specimens was between that of the 2024 and 7075 homogenous specimens. With further crack propagation, the dissimilar crack growth rate gradually approached that of the 2024 homogeneous specimen. This could be attributed to the lower hardness of the 2024 material, which facilitates crack propagation and ultimately allows the crack to extend into the 2024 side.

## 5. Numerical Analysis of Fatigue Crack Growth

**Figure 7:**
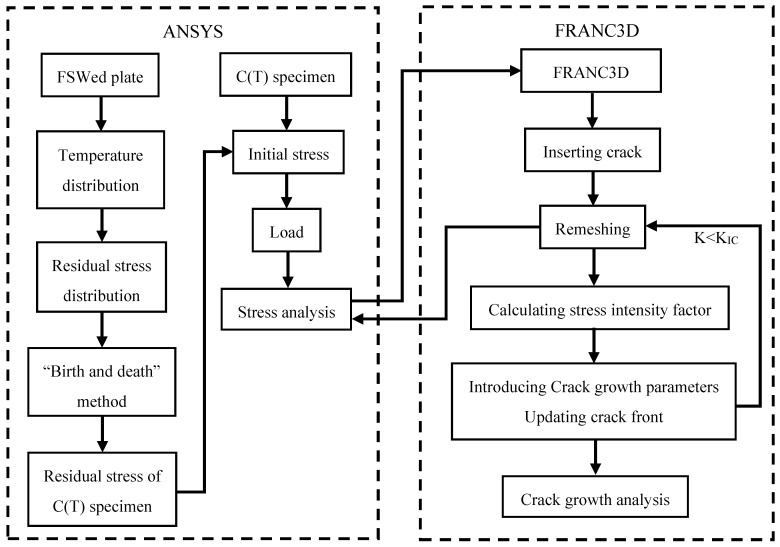
Flow chart of the process for calculating the fatigue crack growth behavior.

The residual stress distribution of the FSWed specimens was calculated using ANSYS software, and the stress results were subsequently imported into FRANC3D for crack growth and post-processing analysis. The calculation process is illustrated in Figure 7.

### 5.1. Residual Stress Distribution of the Fatigue Specimen

**Figure 8:**
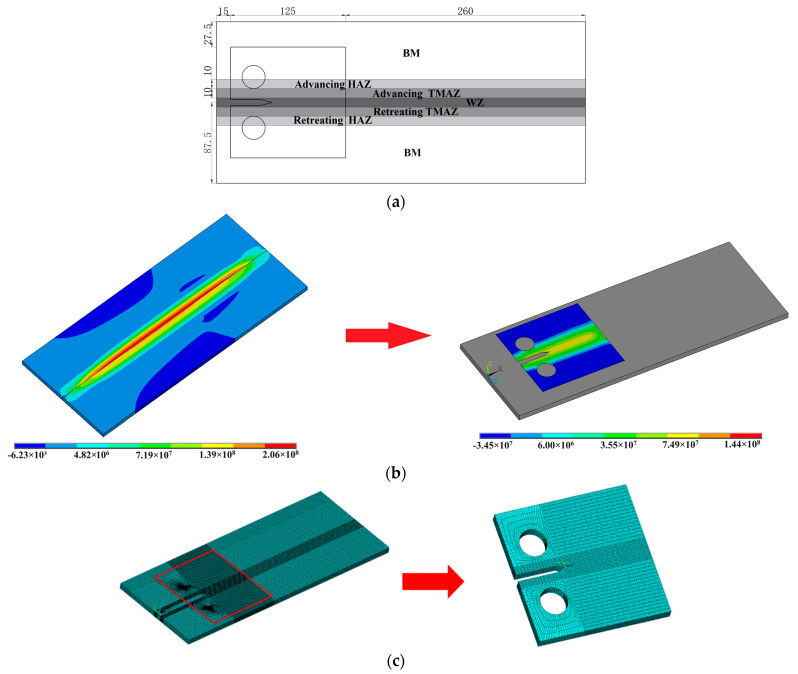
Residual stress model of the C(T) specimen: (**a**) size of the specimen (mm); (**b**) “birth and death element” method (Pa); (**c**) finite element meshing.

The coupled thermo-mechanical model was employed to calculate the residual stress distribution of the welded plates [28]. Considering the influence of the mechanical properties on the fatigue crack growth rate, the model was divided into seven zones, which represent the base metal (BM), the advancing HAZ, the advancing TMAZ, the WZ, the retreating TMAZ, and the retreating HAZ, respectively. The thermo-mechanical properties of the alloys are shown in Table 3, Table 4 and Table 5 [13]. For the 2024/7075 dissimilar welded zone, the parameters were derived as the mean of those for the 2024 and 7075 specimens. Figure 8a shows the size and position of the C(T) specimen. The “birth and death element” method was used to calculate the residual stress of the C(T) specimen (Figure 8b). The FSWed plate model includes 105,992 elements, while the C(T) specimen contains 40,768 elements (Figure 8c).

The rate of heat generation can be obtained using the following equation [30]:

(1) $$\dot{q}=\int_{r_{0}}^{R_{0}}2\pi \omega \cdot r^{2}\mu (T)p(T)dr=\frac{2}{3}\pi \omega \mu (T)p(T)(R_{0}^{3}-r_{0}^{3})$$

**Figure 9:**
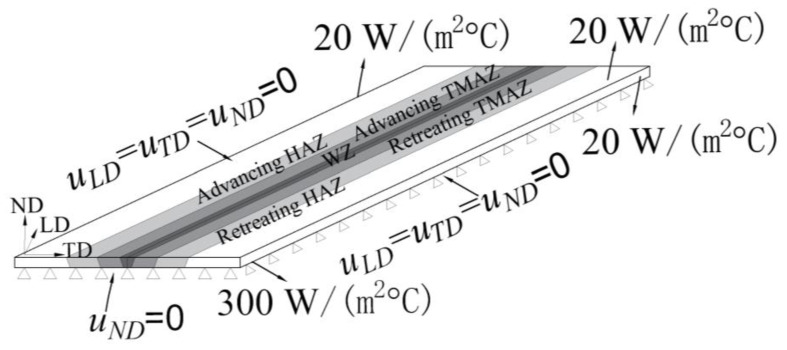
Boundary conditions.

The boundary conditions are illustrated in Figure 9. The surface heat input from the shoulder was applied to the top surface of the specimen with a radius of 10 mm. The volume heat from the pin was generated as a body heat source with dimensions of 5 mm in diameter and 7.5 mm in length. As the temperature varied over time during welding, the coefficient of friction (*μ*) was assumed to be 0.6, and the pressure of the welding tool (*p*) was 10 kN. The ambient temperature was set at 25 °C. The heat transfer coefficient of the top and side surfaces was assumed to be 20 W/(m^2^ °C), while that of the bottom surface was 300 W/(m^2^ °C).

**Figure 10:**
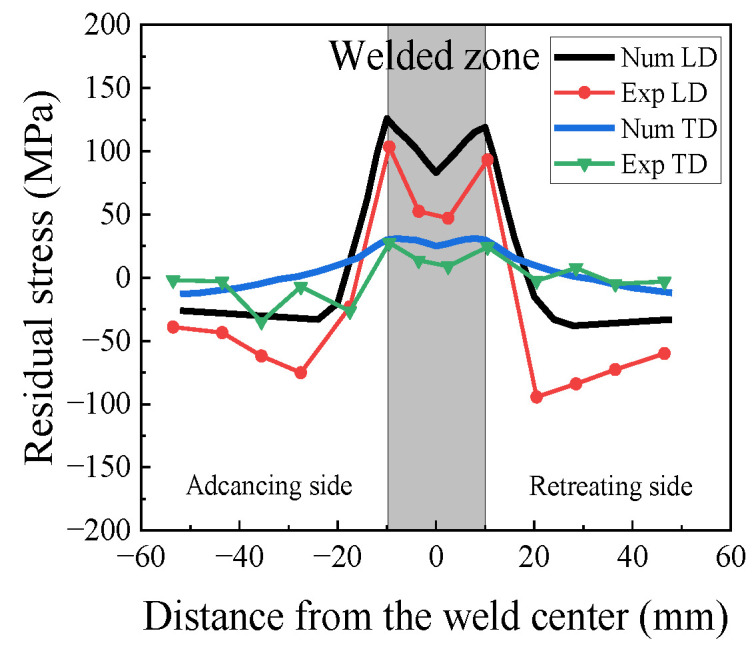
Comparison of the residual stress profiles of the numerical and X-ray diffraction results.

A comparative analysis of the numerical and measured residual stress profiles is shown in Figure 10. The numerical simulation of residual stress also exhibits a “double-peak” pattern. In the advancing TMAZ, the maximum LD stress reaches 126 MPa, which is 22.5 MPa higher than that of the measurements. Moreover, for the TD, the numerical and measured residual stress values are close, with a difference of 5.3 MPa in the retreating TMAZ. It can be seen that the numerical simulation results are in agreement with the X-ray diffraction measurements. Thus, the residual stress model presented in this paper is deemed reliable.

**Figure 11:**
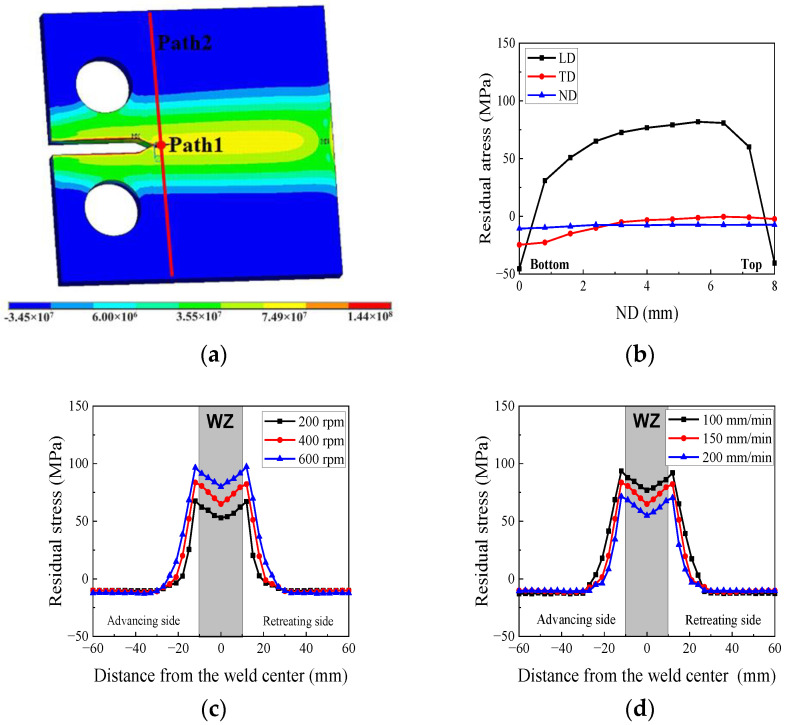
Residual stress of the C(T) specimen: (**a**) longitudinal residual stress distribution of 2024 (400 rpm-150 mm/min) (Pa); (**b**) residual stress in Path1; (**c**) effect of rotational speed on LD residual stress in Path 2; (**d**) effect of welding speed on LD residual stress in Path 2.

The longitudinal residual stress distribution of the 2024 C(T) specimen is shown in Figure 11a. Path 1 represents the normal direction in the middle of the specimen, while Path 2 highlights its crossing path. Upon comparison with the welded plate, it is evident that the residual stress in the C(T) specimen was redistributed after being removed from the welded plate. Figure 11b illustrates that the residual stress along Path 1 decreased from top to bottom. Figure 11c shows the longitudinal residual stress of the 2024 C(T) specimen with different rotational speeds in Path 2. It can be seen that for the same welding speed (150 mm/min), the maximum longitudinal residual stress in Path 2 increased with the increasing rotational speed. The impact of the welding speed on the residual stress of the 7075 was investigated, and the result is displayed in Figure 11d. The maximum longitudinal residual stress in Path 2 decreased with increasing welding speed.

### 5.2. Calculation Results of Fatigue Crack Growth

In this paper, fatigue crack propagation in the FSW joints was designed using the Walker model, which incorporates the effects of residual stress and stress relaxation. The Walker model has a simple form, requires few parameters, and is capable of predicting crack growth during stable propagation. The results of the study in Ref. [24] demonstrated that the Walker model exhibited good consistency with the fatigue crack growth tests.

(2) $$\frac{da}{dN}=C{\left[{\left(1-R\right)}^{m}\Delta K\right]}^{n}$$

where *a* is the crack length; *N* is the number of propagation cycles; $\frac{da}{dN}$ is the crack growth rate; *C* is the coefficient of the Walker model; *m* and *n* are the exponents; and Δ*K* is the stress intensity factor range.

**Figure 12:**
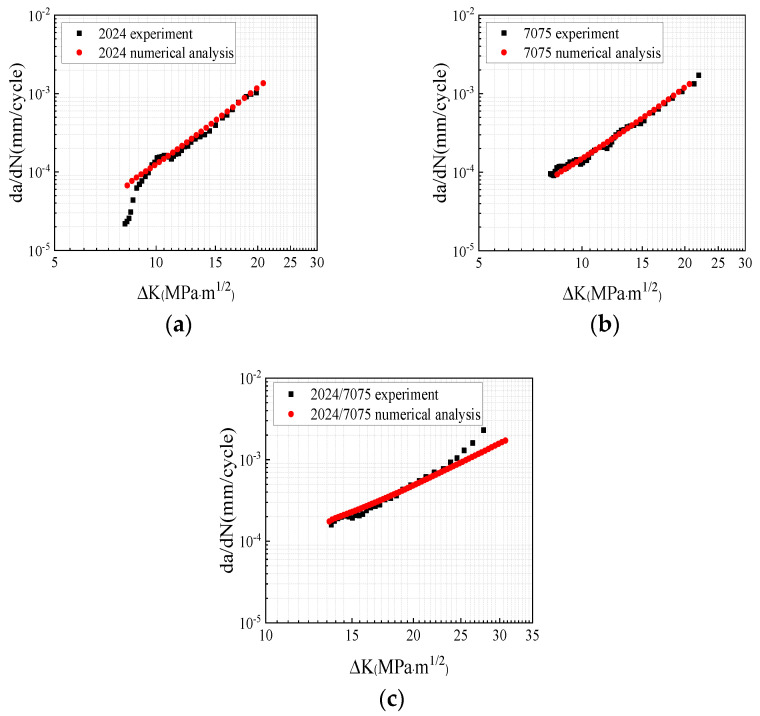
Comparison of the experimental results and numerical analysis: (**a**) 2024 specimen; (**b**) 7075 specimen; (**c**) 2024/7075 dissimilar welded specimen.

According to the crack growth rates of the 2024 and 7075 base metal specimens, the material constants *C*, *m*, and *n* were determined via data analysis and linear fitting (Table 6). For the dissimilar welded specimens, the average values of the material constants from the 2024 and 7075 base metals were utilized. Figure 12 presents the comparison of the fatigue specimens and the results of the numerical calculation, respectively. It is shown that the crack growth predictions were in agreement with the experimental results.

**Figure 13:**
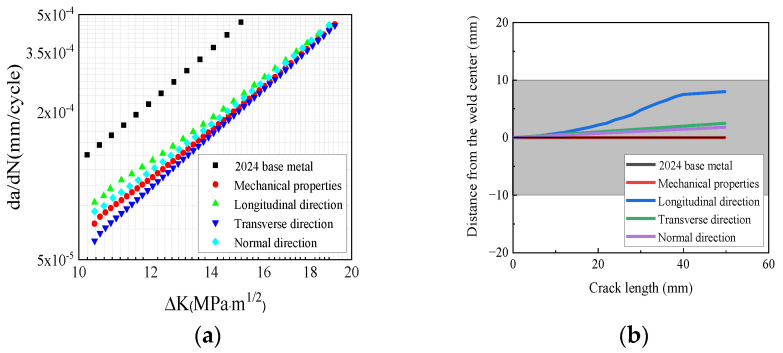
Comparison of the fatigue crack growth rate in the 2024 specimen: (**a**) curve of the fatigue crack growth rate; (**b**) path of the fatigue crack growth.

The residual stress with a rotational speed of 400 rpm and a welding speed of 150 mm/min was applied to the C(T) specimen. The fatigue crack growth model was designed with the same load conditions as those in the experiments. To assess the effects of the mechanical properties and residual stress on fatigue crack growth behavior, the fatigue crack growth rates under the following five conditions were compared. These conditions, respectively, represent 2024 “base metal”, “Mechanical properties” (only considering the mechanical properties of the FSWed specimen), “Longitudinal direction” (only considering the residual stress of the longitudinal direction), “Transverse direction” (only considering the residual stress of the transverse direction), and “Normal direction” (only considering the residual stress of the normal direction). The results shown in Figure 13a demonstrate that the 2024 base metal exhibited the highest fatigue crack growth rate. However, the crack growth rate decreased when the residual stresses of the transverse and normal directions were considered in the calculations. This decrease occurred because of the presence of compressive stress in these two directions, although the stress level was quite low. On the other hand, the tensile stress in the longitudinal direction led to an increase in the crack growth rate.

The comparison of the fatigue crack growth paths is presented in Figure 13b. The crack growth path of the “Mechanical properties” condition aligns with that of the base metal, indicating that the mechanical properties of the FSWed specimen did not influence the fatigue crack deflection. When residual stress was taken into account, all the cracks in the specimen deflected upward. However, the crack deflection angle that resulted from the longitudinal residual stress was larger than that observed in the other directions. This difference can be attributed to the higher residual stress value in the longitudinal direction and the greater stress gradient along the thickness of the specimen as compared to the transverse and normal directions.

**Figure 14:**
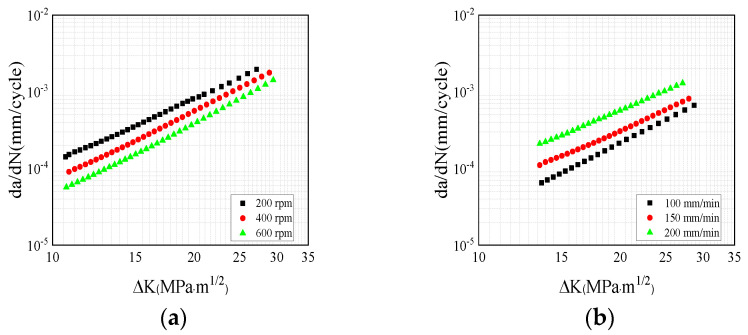
Effect of the welding parameters on fatigue crack growth: (**a**) effect of rotational speed (2024 specimen); (**b**) effect of welding speed (7075 specimen).

The comparison of the fatigue crack growth rates for the 2024 specimen under different rotational speeds is shown in Figure 14a. The results demonstrate that in the 2024 FSWed specimen, the fatigue crack growth rate increased with the increasing rotational speed. This occurred because the residual stress in the longitudinal direction in the 600 rpm specimen was higher than that in the other directions. Thus, the changes in mechanical properties that occurred due to grain refinement in the welded joint were counteracted, which thereby led to an increased rate of crack growth. In Figure 14b, the fatigue crack growth rate of the 7075 specimens decreased with the increasing welding speed. Hence, for the 7075 fatigue specimens, the fatigue crack growth rate was also predominantly influenced by the residual stress.

## 6. Microstructure Analysis

**Figure 15:**
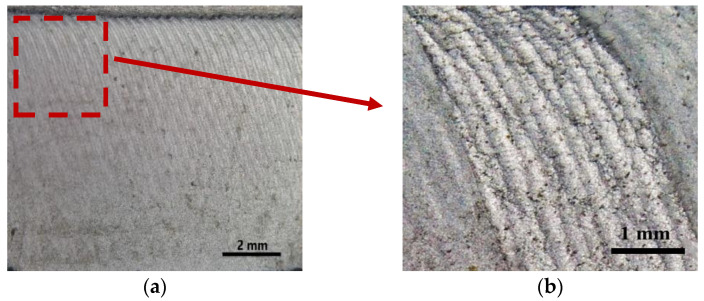
(**a**) Fracture morphology of the 2024 400 rpm-150 mm/min specimen; (**b**) local enlarged image.

The fracture morphologies of the 2024 (400 rpm-150 mm/min) specimens in the fatigue propagation zone are presented in Figure 15. These specimens exhibited fatigue striations. Notably, the crack at the front exhibited an inclination rather than expanding vertically. This phenomenon occurred as the residual stress in the specimen gradually decreased from the top to the bottom, and because the grain size at the bottom of the WZ was smaller than that at the top [13]. As a result, the rate of fatigue crack propagation at the top was faster than that at the bottom.

**Figure 16:**
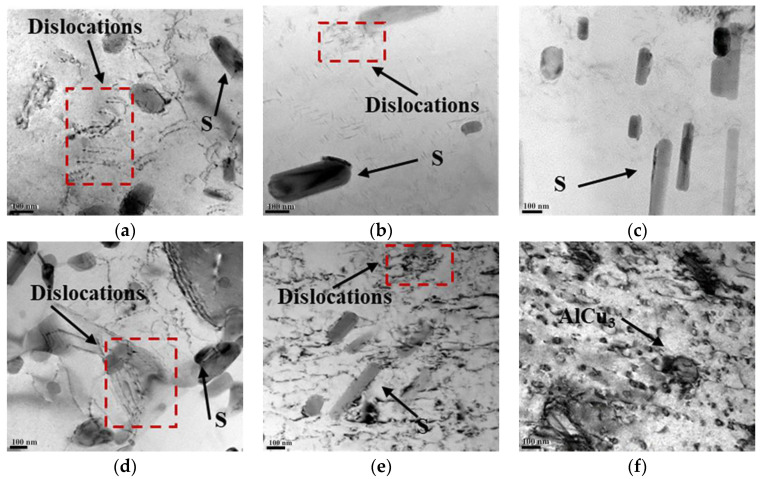
TEM diagrams of the welded joints of the 2024 specimen: (**a**) 400 rpm-150 mm/min WZ; (**b**) 400 rpm-150 mm/min advancing TMAZ; (**c**) 400 rpm-150 mm/min HAZ; (**d**) 200 rpm-150 mm/min WZ; (**e**) 200 rpm-150 mm/min advancing TMAZ; (**f**) base metal.

In Figure 16, the TEM diagrams of the 2024 welded joints with different rotational speeds are shown using the Talos F200X at Xi’an University of Architecture and Technology in Xi’an, China. The TEM images of the WZ confirm the presence of needle-shaped S-phase (Al_2_CuMg) precipitates [31]. In the WZ (Figure 16a,d), most of the dislocations appeared in the form of distortion and entanglement, which resulted from the significant distortion caused by the rotation of the stirring probe. In the TMAZ, the S-phase was also present, with more dislocation entanglement observed around the grain boundaries (Figure 16b,e). This corresponds to the residual stress measurements shown in Figure 2. In the HAZ of the 400 rpm-150 mm/min specimen (Figure 16c), the precipitated S-phase increased due to the influence of the welding thermal cycling, while the extent of dislocation entanglement decreased relatively. In Figure 16f, fewer dislocation cell substructures and AlCu_3_ precipitates (remnants from the aging treatment process) were observed in the base material.

A comparison between the 200 rpm and 400 rpm specimens revealed that the 200 rpm specimen exhibited a higher dislocation density and a greater presence of precipitated phases in the WZ and TMAZ. During the process of fatigue crack propagation, these precipitated phases not only pinned dislocations but also facilitated the generation of additional dislocations, thereby leading to dislocation entanglement. As a result, the dislocation density and sliding resistance increased within the welding joint, which thus resulted in a decreased rate of fatigue crack propagation. This explains why the fatigue crack growth rate of the 200 rpm specimen is lower than that of the 400 rpm from a microstructural perspective, as shown in Figure 5a. Additionally, when compared to the WZ, the S-phase precipitates in the TMAZ and HAZ were coarser; this is a phenomenon that is well-documented in the literature [32,33]. Therefore, the crack predominantly propagated toward the TMAZ, as shown in Figure 4 and Figure 5.

## 7. Conclusions

In this study, the effect of residual stress and microstructure on the fatigue crack growth behavior of 2024-T3 and 7075-T6 friction stir welded joints with different welding parameters was analyzed. The key findings are as follows:

(1) The welding parameters significantly influenced the fatigue crack growth rate. For the 2024 specimens, the fatigue crack growth rate increased with the higher rotational speed, while for the 7075 specimens, it decreased with the higher welding speed.
(2) The longitudinal residual stress had the most significant impact on crack deflection. Additionally, the tensile residual stress in the welded joints accelerated crack growth, partially counteracting the reduction in the fatigue crack growth rate due to grain refinement. Conversely, the compressive residual stress reduced the stress intensity factor, thereby resulting in a diminished crack driving force.
(3) The microstructural characteristic of the welded joints aligns with the distribution of residual stress. It was revealed that the precipitated phase both pinned dislocations and induced the generation of additional dislocations, thereby resulting in dislocation plugging. Consequently, as the rotational speed decreased, the dislocation density and sliding resistance increased, which thus led to a decreased rate of fatigue crack propagation. Furthermore, the TMAZ and HAZ contained coarser precipitates compared to the WZ, causing the crack to deflect towards the TMAZ.

## Figures and Tables

**Table 1:** Chemical compositions of the 2024-T3 and 7075-T6 alloys.

Material	Chemical Composition (%)								
2024-T3	Cu	Si	Fe	Mn	Mg	Zn	Cr	Ti	Al
	3.8–4.9	0.5	0.5	0.3–0.9	1.2–1.8	0.25	0.1	0.15	Base
7075-T6	Si	Fe	Mn	Cu	Mg	Cr	Zn	Ti	Al
	0.4	0.5	0.3	1.2–2.0	2.1–2.9	0.18–0.28	5.1–6.1	0.2	Base

**Table 2:** Welding parameters.

Specimen	Material	Rotational Speed (rpm)	Welding Speed (mm/min)
1	2024 homogeneous	400	150
2	2024 homogeneous	200	150
3	7075 homogeneous	400	150
4	7075 homogeneous	400	100
5	2024/7075 dissimilar	400	150

**Table 3:** Thermo-mechanical properties of the 2024 and 7075 aluminum alloys.

	Temperature (°C)	25	100	200	300	400	500
2024	Specific heat (J/(kg·K))	900	910	950	1020	1100	1100
	Density (kg/m^3^)	2770	2760	2730	2710	2690	2670
	Thermal conductivity (W/(m·K))	126	129	153	178	187	194
	Poisson’s ratio	0.33	0.33	0.33	0.33	0.33	0.33
	Young’s modulus (MPa)	7.2 × 10^4^	7.1 × 10^4^	6.3 × 10^4^	5.3 × 10^4^	4.5 × 10^4^	4.3 × 10^4^
	Coefficient of thermal expansion (×10^−5^/K)	2.11	2.29	2.38	2.47	2.65	2.87
7075	Specific heat (J/(kg·K))	835.4	897	974	1012.5	1128	1205
	Density (kg/m^3^)	2800	2800	2800	2800	2800	2800
	Thermal conductivity (W/(m·K))	114.8	128.4	142.2	152.7	160.8	166.7
	Poisson’s ratio	0.33	0.33	0.33	0.33	0.33	0.33
	Young’s modulus (MPa)	7.3 × 10^4^	7.2 × 10^4^	6.4 × 10^4^	5.6 × 10^4^	4.8 × 10^4^	4.8 × 10^4^
	Coefficient of thermal expansion (×10^−5^/K)	2.3	2.44	2.66	2.87	3.09	3.2

**Table 4:** Mechanical parameters of the 2024 FSWed specimen.

	Temperature (°C)	25	100	200	300	400	500
2024 base metal	Yield stress (MPa)	306	261	152	57	13	5
	Plastic modulus (MPa)	530	400	330	233	95	65
2024 advancing HAZ	Yield stress (MPa)	326	278	162	60	13	5
	Plastic modulus (MPa)	424	289	103	63	35	15
2024 advancing TMAZ	Yield stress (MPa)	205	174	101	38	9	3
	Plastic modulus (MPa)	820	750	600	370	210	90
WZ	Yield stress (MPa)	229	198	113	42	9	3
	Plastic modulus (MPa)	960	870	720	270	105	70
2024 retreating TMAZ	Yield stress (MPa)	211	180	105	39	9	3
	Yield stress (MPa)	820	750	600	370	210	90
7075 retreating HAZ	Yield stress (MPa)	313	267	155	58	13	5
	Plastic modulus (MPa)	424	289	103	63	35	15

**Table 5:** Mechanical parameters of the 7075 FSWed specimen.

	Temperature (°C)	25	100	200	300	400	500
7075 base metal	Yield stress (MPa)	455	389	278	47	33	20
	Plastic modulus (MPa)	250	210	150	50	15	10
7075 advancing HAZ	Yield stress (MPa)	381	326	233	39	27	16
	Plastic modulus (MPa)	300	260	190	90	45	25
7075 advancing TMAZ	Yield stress (MPa)	252	215	154	26	18	11
	Plastic modulus (MPa)	990	870	775	300	145	90
WZ	Yield stress (MPa)	259	221	158	26	18	11
	Plastic modulus (MPa)	940	830	680	230	75	50
7075 retreating TMAZ	Yield stress (MPa)	264	226	161	79	18	11
	Plastic modulus (MPa)	990	870	775	300	145	90
7075 retreating HAZ	Yield stress (MPa)	371	317	226	38	26	16
	Plastic modulus (MPa)	300	260	190	90	45	25

**Table 6:** Parameters of the Walker model.

Material	*C*	*m*	*n*
2024	2.71 × 10^−10^	4.360	3.375
7075	6.79 × 10^−10^	3.957	3.108
2024/7075	4.75 × 10^−10^	4.159	3.242

## Data Availability

Data are contained within the article.
